# Effects of Ammonium Chloride-Mediated Control of Digestive Vacuole Acidification on Endosymbiosis Between *Paramecium tritobursaria* and *Chlorella variabilis*

**DOI:** 10.3390/microorganisms14081742

**Published:** 2026-08-08

**Authors:** Narumi Uchida, Yuuki Kodama

**Affiliations:** 1 Agricultural and Life Sciences, Graduate School of Natural Science and Technology, Shimane University, Matsue-shi 690-8504, Japan; 2 Department of Life Sciences, Faculty of Life and Environmental Sciences, Shimane University, Matsue-shi 690-8504, Japan

**Keywords:** *Paramecium tritobursaria*, *Chlorella variabilis*, endosymbiosis, digestive vacuole, vacuolar acidification, ammonium chloride, lysosomal fusion, symbiosis establishment, perialgal vacuole

## Abstract

*Paramecium tritobursaria* is a ciliate that harbors intracellular *Chlorella* sp. symbionts and serves as a model organism for studying endosymbiosis. After ingestion by *P. tritobursaria*, some algal cells are digested within digestive vacuoles (DVs), whereas others escape digestion and become enclosed by a perialgal vacuole (PV) membrane, establishing a stable symbiosis. We investigated whether inhibiting DV acidification with ammonium chloride (NH_4_Cl) modulates algal digestion and the establishment of endosymbiosis. Congo red-stained yeast assays showed that treatment with 20 mM NH_4_Cl for 40 min effectively suppressed DV acidification. Under these conditions, algal intracellular behavior was altered; escape from DVs appeared to be delayed, particularly at 6 h after uptake, and many cells remained within the DVs. Despite this delay, symbiosis was established at 24 h in both groups. Notably, NH_4_Cl treatment significantly increased the symbiosis establishment rate, with an average 1.5-fold increase and up to a 3-fold increase compared with the control. These results demonstrate that transient inhibition of DV acidification alters digestion dynamics and promotes the establishment of symbiosis. This study provides a novel strategy for dissecting *P. tritobursaria*–*Chlorella* sp. endosymbiosis through controlled modulation of DV maturation.

## 1. Introduction

*Paramecium tritobursaria* is a ciliate that harbors green algae of the genus *Chlorella* as intracellular endosymbionts and has been widely used as a model organism for studying intracellular endosymbiosis. The relationship between *P. tritobursaria* and its symbiotic *Chlorella* spp. is mutualistic, in which the host supplies nitrogen sources and carbon dioxide to the symbionts, whereas the symbiotic algae provide the host with oxygen and photosynthetically produced carbohydrates [1,2,3]. This symbiotic system is particularly useful for analyzing the cellular mechanisms of endosymbiosis because the host and symbionts can be experimentally separated and re-established [4,5,6,7,8,9,10,11].

*Chlorella* is one of the most well-known genera of microalgae, traditionally characterized by spherical to oval unicellular morphology and reproduction by autospores [12]. To date, approximately 100 species have been described [13], but only a limited number are capable of establishing stable intracellular endosymbiosis with *P. tritobursaria*. *Chlorella vulgaris*, *Parachlorella kessleri* (formerly *Chlorella kessleri*), *C. sorokiniana*, and *C. variabilis* have been reported as species that successfully infect alga-free *P. tritobursaria* under experimental conditions [14,15,16]. Among these, the *P. kessleri* strain NIES-2152 exhibits remarkable metabolic plasticity, with a high capacity for starch and triacylglycerol accumulation, depending on the culture conditions. Under nutrient-rich conditions, cells remain photosynthetically active without accumulating storage compounds, whereas nutrient stress, such as sulfur deprivation, induces starch accumulation followed by lipid production [17]. These storage compounds are of considerable interest as feedstocks for biofuel and chemical production [18,19]. Notably, recent work indicates that such metabolically flexible strains can establish long-term stable symbiosis with *P. tritobursaria*, and their metabolic state may influence symbiosis efficiency [20]. These findings suggest that not only species identity but also the physiological and metabolic state of *Chlorella* sp. play a crucial role in determining the success of symbiosis, highlighting the potential of the *P. tritobursaria*–*Chlorella* sp. system as both a model for endosymbiosis and a platform for biotechnological applications. However, despite these advances in understanding algal factors, the role of host cellular processes, particularly digestive vacuole (DV) dynamics, in determining the fate of ingested algae remains poorly understood.

During the establishment of *Chlorella* sp. endosymbiosis in *P. tritobursaria*, ingested algal cells are first internalized through the host cytopharynx and enclosed within a DV. The DV then undergoes membrane fusion with acidosomes, resulting in rapid vacuolar acidification, followed by fusion with lysosomes containing digestive enzymes, thereby initiating digestion. Acidosomal fusion occurs approximately 0.5 min after DV formation, whereas lysosomal fusion occurs at approximately 2–3 min. After acidosomal fusion, the pH inside the DV decreases to approximately 2.5–3.0, forming an extremely acidic environment. Subsequently, following lysosomal fusion, the vacuolar pH recovers toward near-neutral conditions [21]. Despite these harsh conditions, a subset of algal cells avoids digestion and is ultimately enclosed by a perialgal vacuole (PV) membrane, a specialized type of symbiosome membrane that does not fuse with lysosomes. Being enclosed within the PV membrane allows algae to persist beneath the host cell cortex and establish stable endosymbiosis [21,22,23,24].

Previous studies have demonstrated that the ability of symbiotic algae to avoid digestion in host DVs is strongly influenced by light conditions. When isolated algae are maintained under constant light, many algal cells acquire resistance to host lysosomal enzymes, whereas algae kept under dark conditions are almost completely digested after ingestion. Importantly, this resistance is not rescued by the addition of maltose and is not inhibited by a photosynthesis inhibitor, suggesting that light-dependent but yet unidentified physiological factors are required [25]. In addition, the PV surrounding each symbiotic alga is maintained under acidic conditions, which is thought to facilitate algal efficient photosynthesis-dependent carbon transfer, as maltose release is enhanced at low pH and is driven by a proton gradient [26,27]. In contrast, acidification of DVs promotes the degradation of ingested particles, including *Chlorella* sp. cells. Thus, vacuolar acidification may play fundamentally different roles depending on the compartment, supporting symbiosis in the PV while promoting digestion in the DV. However, the effects of experimental manipulation of DV acidification on algal fate and the establishment of symbiosis have not been systematically investigated.

In a classical study using another *Paramecium* species, *P. multimicronucleatum*, inhibition of DV acidification by ammonium chloride (NH_4_Cl) was shown not only to suppress lysosomal fusion but also to markedly reduce the efficiency of subsequent digestive processes [28]. Detailed analyses revealed that both the extent and rate of lysosome–DV fusion are closely correlated with the rate of DV acidification, indicating that acidification is not merely a consequence of DV maturation but a key regulatory factor governing lysosomal interactions. Furthermore, experimental manipulation of the pH of the DV demonstrated that inhibition of acidification at early stages significantly reduced lysosome binding and fusion, whereas perturbation after acidification had a much weaker effect. In addition, cytochemical studies using latex bead-containing DVs showed that acid phosphatase activity, a marker of lysosomal enzymes, was detected only after vacuole maturation had progressed, indicating that lysosomal fusion occurs only after the initial acidification phase [29]. These findings highlight the importance of both the timing and magnitude of acidification in controlling DV maturation and its function. Together, these findings suggest that DV acidification is an important regulatory factor governing DV maturation and digestive activity. Although NH_4_Cl is widely used to elevate lysosomal and endosomal pH, its effects on DV maturation and algal endosymbiosis in *P. tritobursaria* remain unclear. Given the critical role of DV acidification in regulating lysosomal fusion, this process may also be directly relevant to the establishment of symbiosis, where suppression of algal digestion is required. To our knowledge, no previous study has directly examined whether experimental manipulation of DV acidification affects the establishment of endosymbiosis in *P. tritobursaria*. Accordingly, the specific contribution of DV acidification to the balance between algal digestion and symbiosis establishment remains unknown. We hypothesized that transient inhibition of DV acidification would alter the intracellular fate of ingested algae and thereby affect the efficiency of symbiosis establishment. In this study, we first examined the conditions under which NH_4_Cl inhibits DV acidification in *P. tritobursaria* using budding yeast cells stained with pH-sensitive dye. We then performed symbiosis experiments with isolated *Chlorella* sp. under the same conditions to elucidate how modulation of DV acidification influences whether ingested algal cells are digested or successfully establish endosymbiosis. By combining pharmacological manipulation of DV acidification with experimental reinfection assays, this study provides a new experimental framework for investigating how host digestive processes influence the transition of ingested *Chlorella* sp. cells from prey to stable endosymbionts.

## 2. Materials and Methods

### 2.1. Strains and Cultures

The cells used in this study were identified as *P. tritobursaria* according to the classification of Spanner et al. (2022) [30], corresponding to *P. bursaria* syngen 1 in the classification of Bomford (1966) [31]. Two *P. tritobursaria* strains (mating type I) were used in this study: the alga-free (aposymbiotic) strain Yad1w and the algae-bearing strain Yad1g1N. The Yad1g1N strain was originally established by infecting Yad1w cells with the symbiotic *C. variabilis* strain 1N [32]. *P. tritobursaria* cells were cultured in red pea (*Pisum sativum*) extract medium [33] prepared in modified Dryl’s solution (MDS), in which NaH_2_PO_4_·2H_2_O was replaced with KH_2_PO_4_ [34]. The bacterium *Klebsiella aerogenes* (ATCC 35028) was added as a food source to the red pea extract medium [35]. Several hundred *P. tritobursaria* cells were used to inoculate 2 mL aliquots of culture medium in test tubes, followed by daily addition of 2 mL of fresh medium for 12 days. Cultures were maintained at 23 ± 1 °C and used for experiments 2 days after the final feeding, corresponding to the early stationary phase of growth. Yad1g1N cells were cultured under constant illumination (24L:0D) using fluorescent light (27 W, natural white) at an intensity of 20–30 μmol photons m^−2^ s^−1^.

*Saccharomyces cerevisiae* strain BY137 was obtained from NBRP Yeast (https://yeast.nig.ac.jp/yeast/top.xhtml (accessed on 4 August 2026)) and used in this study. Yeast cells were cultured on YPD agar slant medium prepared in test tubes containing 1% yeast extract, 2% casein peptone, 2% D-glucose, and 1.5% agar in ultrapure water.

### 2.2. Staining Yeast Cells with Congo Red

To evaluate the acidification of DVs in alga-free *P. tritobursaria*, budding yeast cells were stained with 0.5% Congo red. Yeast cells were collected using a disposable loop and suspended in 1 mL of ultrapure water in a 1.5 mL microcentrifuge tube. The cell suspension was centrifuged at 4500× *g* for 1 min, and the supernatant was removed. The yeast pellet was resuspended in 0.5% (*w*/*v*) Congo red dissolved in ultrapure water to a final volume of 1 mL. The suspension was mixed thoroughly by pipetting. Staining was performed by heating the suspension at 100 °C for 15 min using a dry block incubator (BSR-M002, BSR-MiniTC, Bio Medical Science Co., Ltd., Tokyo, Japan). After staining, the cells were centrifuged at 4500× *g* for 1 min, and the supernatant was removed. The pellet was washed with ultrapure water and resuspended to a final volume of 1 mL; this washing step was repeated three times. Finally, the stained yeast cells were resuspended in 1 mL of MDS and maintained at 22 ± 1 °C until use. Hereafter, these cells are referred to as stained yeast.

### 2.3. Inhibition of DV Acidification in Alga-Free P. tritobursaria by NH_4_Cl

The concentration of NH_4_Cl (20 mM) was selected based on preliminary experiments evaluating the effects of NH_4_Cl on *P. tritobursaria* cell viability and was used as a practical concentration for transient inhibition of DV acidification. A 3 mL suspension containing alga-free *P. tritobursaria* (5 × 10^3^ cells/mL) and stained yeast cells (5 × 10^7^ cells/mL) was prepared and incubated in the presence of NH_4_Cl (final concentration 20 mM) for 1.5 min. After incubation, NH_4_Cl and non-ingested yeast cells were removed by washing the cells on a 15 µm nylon mesh with 100 mL of MDS. The *P. tritobursaria* cells were then resuspended in a solution containing 20 mM NH_4_Cl and further incubated for 40 min. In some experiments, NH_4_Cl was removed after 40 min, and *P. tritobursaria* cells were subsequently incubated for an additional 40 min in the absence of NH_4_Cl. In the control experiments, MDS was used instead of NH_4_Cl. Changes in the color of yeast cells internalized within DVs were observed using a differential interference contrast (DIC) microscope (BX53; EVIDENT, Tokyo, Japan). DV acidification was assessed based on the color change of the internalized stained yeast cells from red to blue.

### 2.4. Isolation of Symbiotic C. variabilis from Algae-Bearing P. tritobursaria

To isolate symbiotic *C. variabilis* strain 1N cells, 300 mL of culture of algae-bearing *P. tritobursaria* Yad1g1N was first filtered through two layers of KimWipes to remove gross debris. The filtrate was then passed through a 15 µm nylon mesh, and the retained cells were washed on the mesh with 50 mL of MDS. The washed cells were collected using hand-operated centrifugation (UKG-2; Uchida Rikakiki, Tokyo, Japan) and resuspended in 1 mL of MDS. Phenylmethylsulfonyl fluoride (Sigma-Aldrich, St. Louis, MO, USA) was added to the cells at a final concentration of 0.1 mM, and the cells were homogenized on ice using a Teflon homogenizer (30 strokes) (AS ONE Corporation, Osaka, Japan). The homogenate was filtered through a 15 μm nylon mesh and centrifuged at 4500× *g* for 1 min. The pellet was washed three times with MDS and finally resuspended in 500 μL of MDS. The algal cell density was determined using a Thoma hemocytometer (AS ONE Corporation, Osaka, Japan).

### 2.5. Reinfection of Alga-Free P. tritobursaria with C. variabilis Under NH_4_Cl Treatment

A 3 mL suspension containing alga-free *P. tritobursaria* cells (5 × 10^3^ cells/mL) and isolated *C. variabilis* cells (5 × 10^7^ cells/mL) was prepared and incubated in the presence of 20 mM NH_4_Cl for 1.5 min. Non-ingested algal cells were removed by washing the cells on a 15 µm nylon mesh with 100 mL of MDS. The cells were then resuspended in MDS containing 20 mM NH_4_Cl and further incubated for a total of 40 min from the start of mixing. After 40 min, the NH_4_Cl was removed by washing the cells retained on a 15 µm nylon mesh with 100 mL of MDS. The *P. tritobursaria* cells were then resuspended in MDS and incubated at 22 ± 1 °C for 24 h. Following incubation, the cells were fixed by adding an equal volume of 8% paraformaldehyde to achieve a final concentration of 4% before DIC microscopy. The experiment was conducted with six independent biological replicates. The proportion of cells containing *Chlorella* sp. within the cytoplasm was calculated as the symbiosis establishment rate.

### 2.6. Statistical Analysis

All statistical analyses were performed using R software (version 4.1.3). Differences in the proportions of cells containing acidified DVs were evaluated using Fisher’s exact test. The 95% confidence intervals for these proportions were calculated using the exact binomial method. Differences in symbiosis establishment rates were evaluated using the Mann–Whitney U test. Statistical significance was defined as *p* < 0.001 for Fisher’s exact test and *p* < 0.05 for the Mann–Whitney U test.

## 3. Results

### 3.1. Selection of a pH Indicator for Monitoring DV Acidification

Congo red is a pH indicator that appears blue under acidic conditions and red under neutral-to-alkaline conditions. Therefore, DV acidification in alga-free *P. tritobursaria* was monitored by assessing color changes in Congo red-stained yeast cells in the DV, as described previously [21]. In addition to Congo red, bromophenol blue and bromocresol green were also tested. These indicators turn yellow under acidic conditions and blue under neutral-to-alkaline conditions. However, under our experimental conditions, yeast cells treated with these dyes did not exhibit sufficiently distinct coloration or pH-dependent color changes to allow reliable assessment of DV acidification (Figure S1). Therefore, Congo red was used in all subsequent experiments in this study. Although stained yeast cells can be stored at −20 °C in 1 mL of ultrapure water, prolonged storage reduces both the uptake efficiency and pH-dependent color changes. Therefore, stained yeast cells were used immediately after preparation.

### 3.2. Inhibition of DV Acidification by NH_4_Cl

To examine whether NH_4_Cl inhibits DV acidification, alga-free *P. tritobursaria* cells were allowed to ingest Congo red-stained yeast cells, followed by treatment with 20 mM NH_4_Cl. Figure S2 shows a schematic overview of the experimental design. After 1.5 min of uptake, the non-ingested yeast cells and NH_4_Cl were removed by washing. The cells were then divided into two groups: one continuously treated with NH_4_Cl for up to 80 min, and another in which NH_4_Cl was removed after 40 min. Control cells were treated with MDS instead of NH_4_Cl.

**Figure 1:**
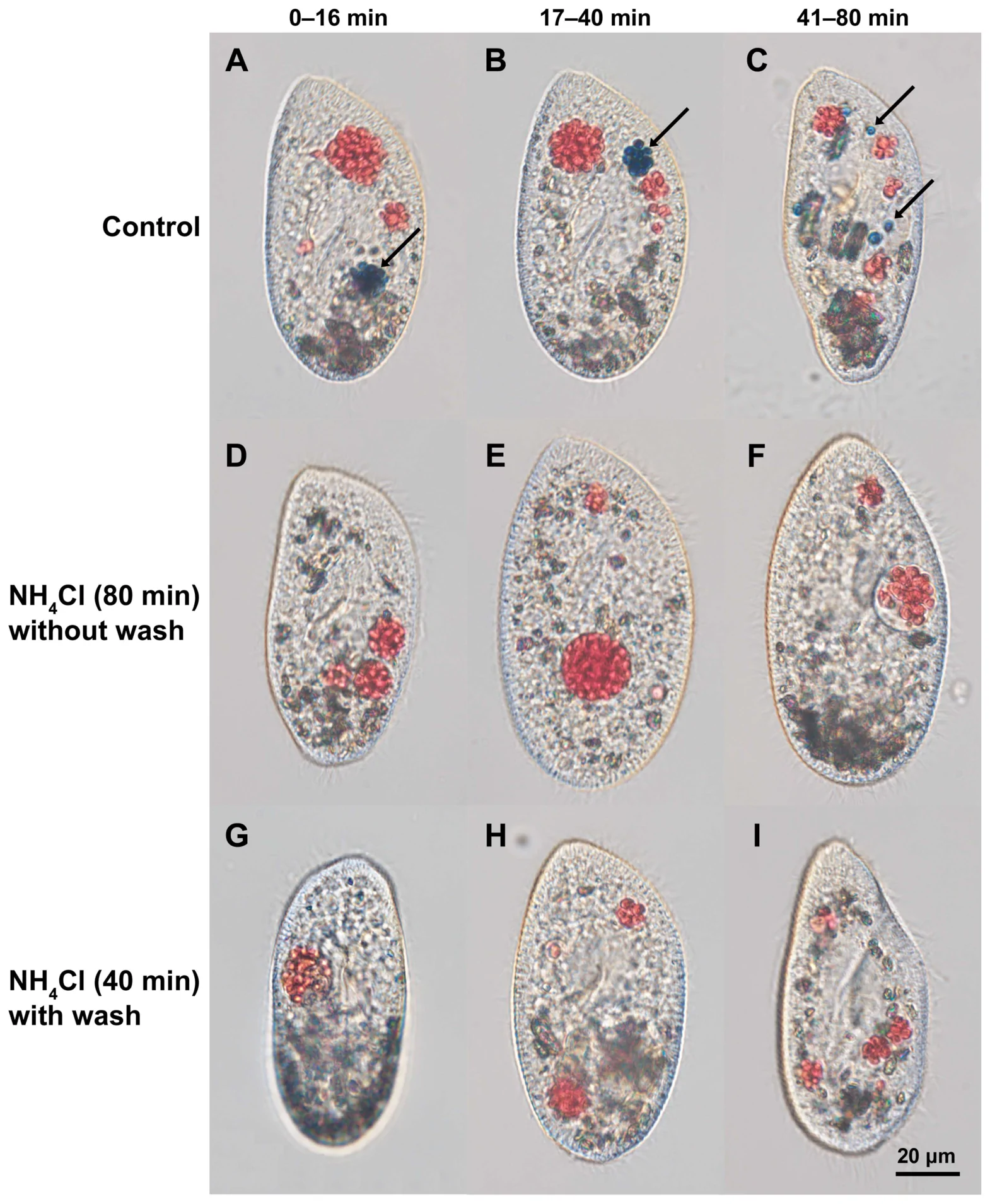
Inhibition of DV acidification by NH_4_Cl in *P. tritobursaria*. DIC images of cells after ingestion of Congo red-stained yeast. Panels (**A**–**C**) show the control cells at 0–16, 17–40, and 41–80 min, respectively. Panels (**D**–**F**) and (**G**–**I**) show the NH_4_Cl-treated cells under two different conditions: treatment for up to 80 min (without wash) and 40 min treatment with subsequent removal of NH_4_Cl (with wash), respectively. Panels (**D**–**F**) and (**G**–**I**) correspond to the same time intervals as panels (**A**–**C**). Acidified DVs are indicated by blue-stained yeast cells (arrows). Scale bar: 20 μm.

In control cells, DV acidification was typically observed within 5–15 min after uptake, as indicated by the color change of stained yeast cells from red to blue (Figure 1A–C). The number and intensity of blue-stained DVs increased over time, indicating progressive acidification during the digestion process. In contrast, the NH_4_Cl-treated cells showed almost no acidified DVs throughout the 80 min observation period, regardless of whether NH_4_Cl was removed after 40 min or was continuously applied (Figure 1D–I). Most ingested yeast cells remained red, suggesting that DV acidification was effectively blocked under these conditions.

**Figure 2:**
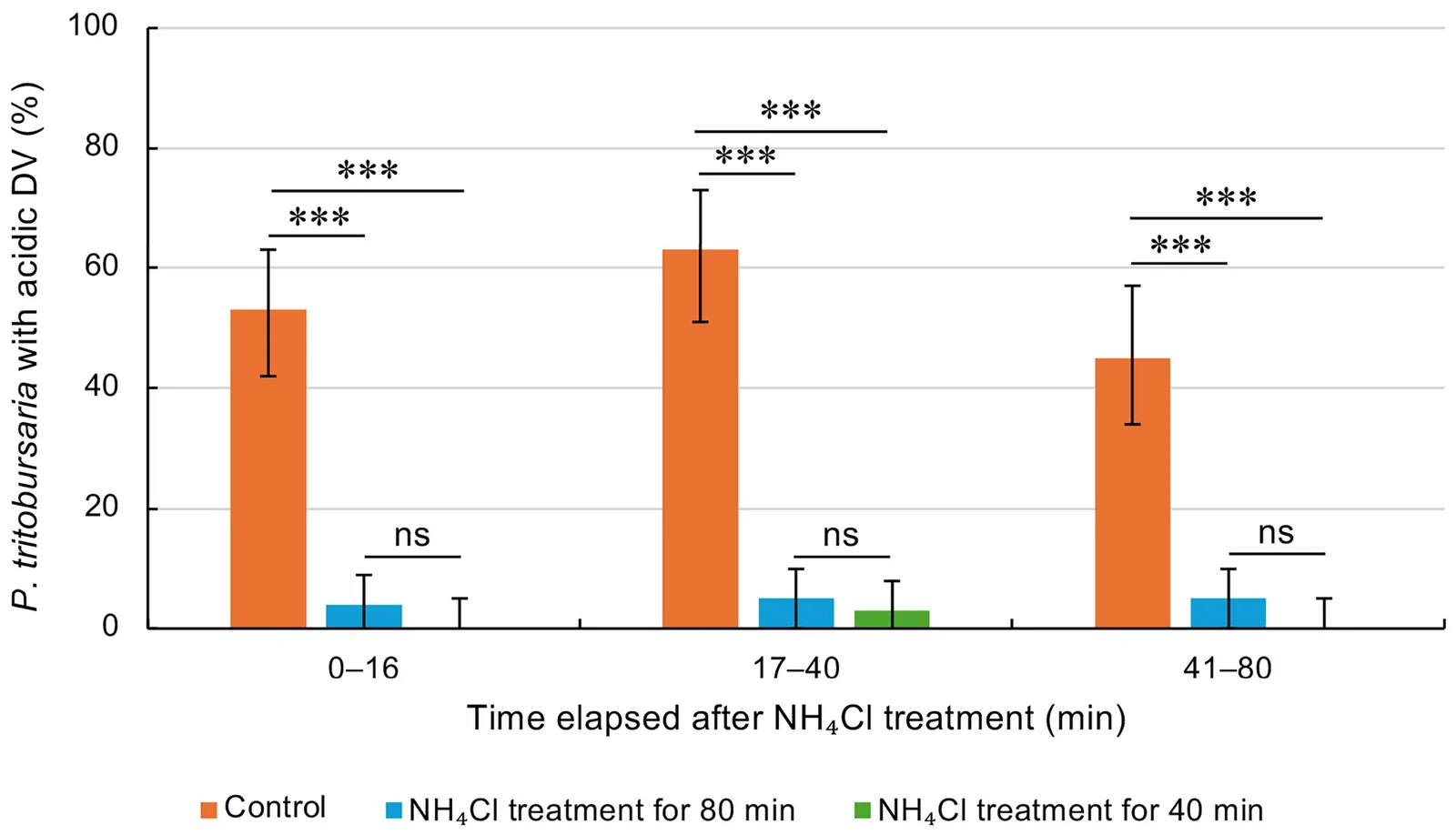
The percentage of *P. tritobursaria* containing acidified DVs was determined during three observation periods (0–16, 17–40, and 41–80 min). Both NH_4_Cl-treated groups showed significantly lower values than the control cells at all time intervals. Error bars indicate the 95% confidence intervals. The data were pooled from three independent experiments. *** *p* < 0.001 (Fisher’s exact test); ns, not significant. Exact *p*-values were as follows: 0–16 min, Control vs. NH_4_Cl (80 min), *p* = 4.71 × 10^−10^; Control vs. NH_4_Cl (40 min), *p* = 6.48 × 10^−11^. 17–40 min, Control vs. NH_4_Cl (80 min), *p* = 1.10 × 10^−15^; Control vs. NH_4_Cl (40 min), *p* = 2.09 × 10^−19^. 41–80 min, Control vs. NH_4_Cl (80 min), *p* = 1.59 × 10^−11^; Control vs. NH_4_Cl (40 min), *p* = 2.35 × 10^−10^.

Acidification occurred rapidly in the control cells and was observed in a large proportion of the cells. Quantitative analysis confirmed these observations: the proportion of cells containing acidified DVs increased and reached a maximum during the 17–40 min interval in control cells, whereas it remained low in both NH_4_Cl-treated groups (Figure 2). At all time intervals examined (0–16, 17–40, and 41–80 min), both NH_4_Cl-treated groups showed significantly lower values than the control group (Fisher’s exact test, *p* < 0.001). No significant difference was detected between the NH_4_Cl 40 min and 80 min treatment groups, suggesting that the removal of NH_4_Cl after 40 min was insufficient to restore acidification within the observation period. Together, these results demonstrate that NH_4_Cl effectively and persistently inhibits DV acidification in alga-free *P. tritobursaria*. The raw data are presented in Table S1.

### 3.3. Effects of Acidification Inhibition on Establishment of Symbiosis

To investigate whether the inhibition of DV acidification affects the establishment of symbiosis, alga-free *P. tritobursaria* cells were allowed to ingest isolated *Chlorella* sp. cells in the presence of 20 mM NH_4_Cl. Figure S3 shows a schematic overview of the experimental design. After 1.5 min of uptake, the non-ingested algal cells and NH_4_Cl were removed by washing, followed by an additional 40 min of incubation with NH_4_Cl. The cells were then cultured for 24 h in the absence of NH_4_Cl. The control cells were treated under identical conditions without NH_4_Cl.

**Figure 3:**
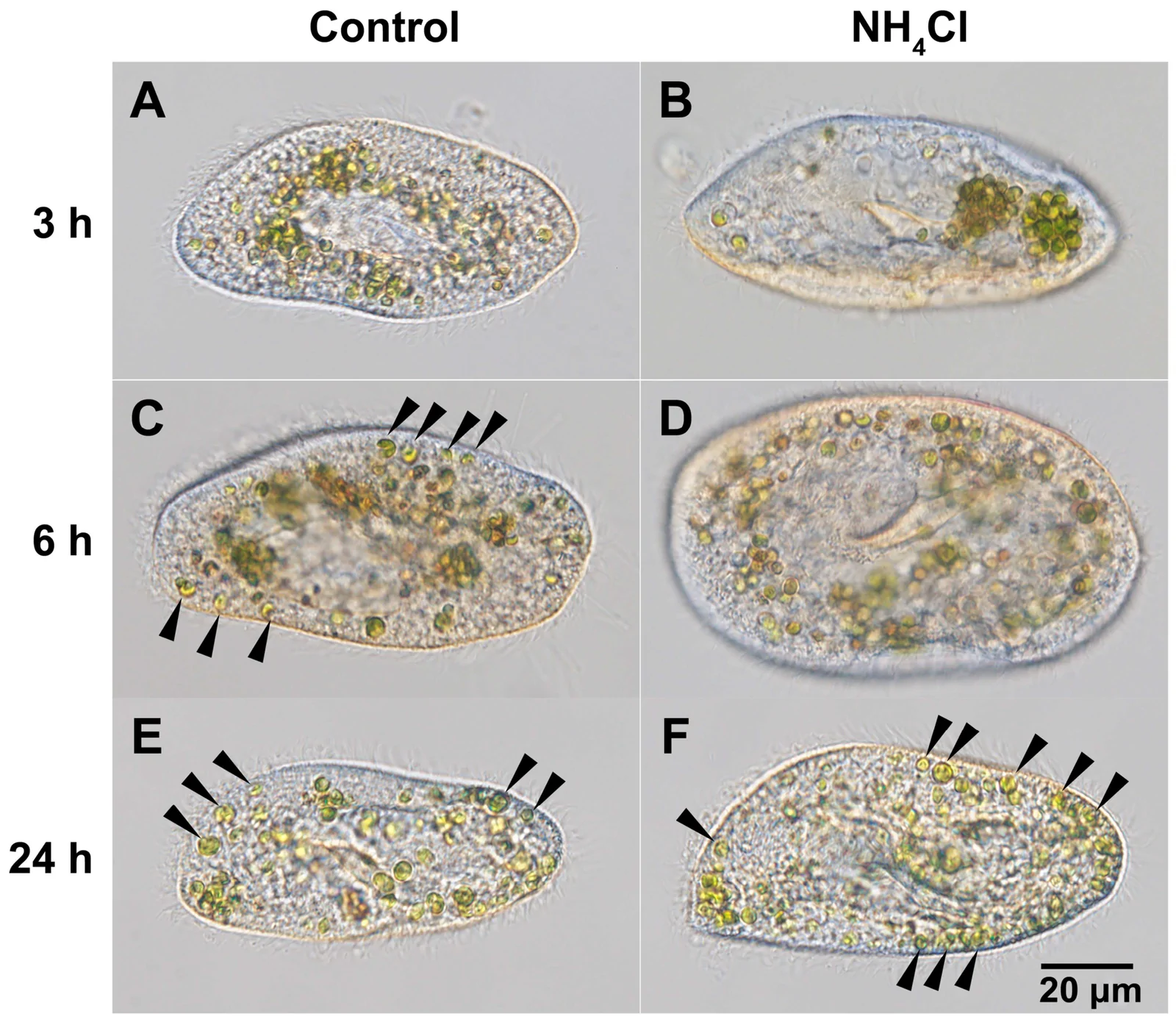
Effects of NH_4_Cl treatment on the intracellular behavior of *Chlorella* sp. DIC images of *P. tritobursaria* cells after algal ingestion. The left column shows the control cells (**A**,**C**,**E**), and the right column shows the NH_4_Cl-treated cells (**B**,**D**,**F**) at 3, 6, and 24 h after uptake. At 6 h, several algal cells were observed as single cells attached just beneath the host cell cortex in the control cells ((**C**) arrowheads), whereas such cells were rarely observed in the NH_4_Cl-treated cells (**D**). At 24 h, such cells were observed in both groups ((**E**,**F**) arrowheads). In both groups, some algal cells appeared brown at early time points (**A**–**D**), indicating ongoing digestion. Scale bar: 20 μm.

At 3 h after uptake, only subtle differences in the intracellular distribution of *Chlorella* sp. cells were observed between the control and NH_4_Cl-treated groups (Figure 3A,B). In NH_4_Cl-treated cells, algal cells tended to remain clustered within DVs, whereas a more dispersed distribution was often observed in control cells. However, clear differences were observed 6 h after uptake (Figure 3C,D). In control cells, some undigested *Chlorella* sp. cells appeared to escape from DVs into the cytoplasm and were observed as single cells attached just beneath the host cell cortex (arrowheads in Figure 3C), indicating the initiation of normal symbiosis establishment, as shown in [21]. In contrast, in NH_4_Cl-treated cells, a larger proportion of algal cells remained confined within DVs, and *Chlorella* sp. cells attached singly beneath the cortex were rarely observed (Figure 3D). The presence of brown, degraded algal cells indicated that the digestive processes were not completely inhibited. By 24 h after uptake, many *Chlorella* sp. cells were observed as single cells attached just beneath the host cell cortex in both control and NH_4_Cl-treated cells (arrowheads in Figure 3E,F), indicating that endosymbiosis had been successfully established.

**Figure 4:**
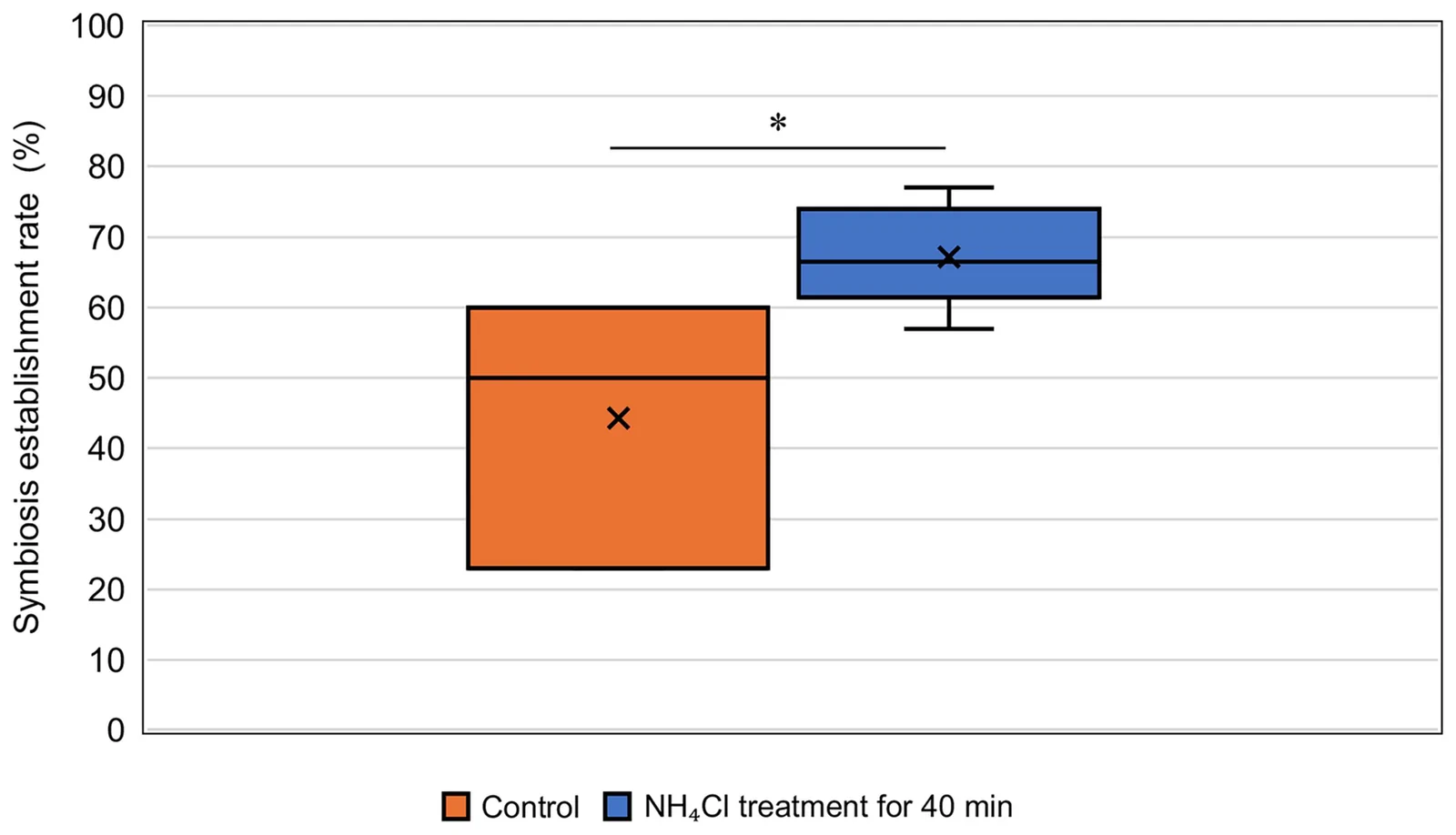
Effect of NH_4_Cl treatment on symbiosis establishment. The symbiosis establishment rate was defined as the percentage of *P. tritobursaria* cells containing *Chlorella* sp. in the cytoplasm 24 h after algal uptake. Data represent six independent experiments (n = 30 cells per experiment). NH_4_Cl-treated cells showed a significantly higher symbiosis establishment rate than control cells (Mann–Whitney U test, *p* = 0.013). Boxes indicate the interquartile range (IQR), the horizontal line indicates the median, whiskers indicate the minimum and maximum values, and crosses indicate the mean values. * *p* < 0.05.

At 24 h after uptake, *P. tritobursaria* cells containing *Chlorella* sp. in the cytoplasm were observed in both groups, indicating that establishment of symbiosis can occur under both conditions. Notably, the retention of algal cells within DVs during the early stages in NH_4_Cl-treated cells suggests that reduced acidification may prolong the residence time of ingested algae within DVs. Quantitative analysis revealed that the symbiosis establishment rate at 24 h was significantly higher in NH_4_Cl-treated cells than in control cells (Mann–Whitney U test, *p* = 0.013) (Figure 4). Across six independent experiments, the symbiosis establishment rate in NH_4_Cl-treated cells was, on average, approximately 1.5-fold higher than that in control cells and reached up to approximately 3-fold higher in some cases. This increase suggests that the suppression of early digestive processes may enable more ingested algae to survive the early stages of intracellular processing and proceed through the symbiosis establishment process. Together, these results indicate that the inhibition of DV acidification enhances the establishment of symbiosis between *P. tritobursaria* and *Chlorella* sp. and suggest that acidification-dependent early digestion constitutes an important barrier to symbiosis establishment. The raw data are presented in Table S1.

## 4. Discussion

### 4.1. Inhibition of DV Acidification by NH_4_Cl and Its Impact on DV Maturation

In this study, we demonstrated that treatment with NH_4_Cl strongly suppressed DV acidification in alga-free *P. tritobursaria* (Figure 1 and Figure 2). Even when NH_4_Cl was removed after 40 min, acidified DVs were rarely observed for at least an additional 40 min. In contrast, the control cells exhibited rapid acidification, which was typically observed within 5–15 min after ingestion. These results are consistent with previous observations in *P. multimicronucleatum*, in which NH_4_Cl inhibits DV acidification [28]. To our knowledge, this is the first study to demonstrate the pharmacological inhibition of DV acidification in *P. tritobursaria*, providing a novel experimental framework for investigating vacuole maturation in this symbiotic ciliate. NH_4_Cl is widely used as an inhibitor of lysosomal acidification; however, its precise mechanism of action remains incompletely understood. In addition to neutralizing luminal pH through its weak base properties, NH_4_Cl has been reported to affect vesicular trafficking and membrane fusion processes [36]. Thus, the observed effects may reflect not only luminal pH neutralization but also broader perturbations in membrane trafficking and vacuole maturation processes. Accordingly, it is difficult to determine whether the inhibition of DV acidification in this study was solely due to pH elevation or whether it also involved alterations in vacuolar dynamics. Interestingly, this inhibitory effect persisted even after the removal of NH_4_Cl, suggesting that ammonium ions transiently accumulate within the cell and interfere with proton transport systems, such as V-ATPases, or their regulation. However, the recovery of intracellular processes observed in subsequent symbiosis experiments indicates that NH_4_Cl-induced inhibition is reversible and does not permanently disrupt the host cellular machinery.

### 4.2. Role of DV Acidification in Regulating Intracellular Behavior of Chlorella sp.

Inhibition of DV acidification significantly altered the intracellular behavior of ingested *Chlorella* sp. In NH_4_Cl-treated cells, a large proportion of algae remained confined within DVs at 6 h after ingestion, whereas in control cells, many algae had already escaped into the cytoplasm and were localized beneath the host cortex (Figure 3). These findings strongly suggest that DV acidification plays a regulatory role in the early intracellular fate of *Chlorella* sp. Acidification of phagosomes and DVs is known to function not only in digestion but also as a key regulatory signal in host–microbe interactions. For example, in the endonuclear symbiont *Holospora obtusa* of *Paramecium caudatum*, DV acidification is required for successful infection of the host’s macronucleus. Inhibition of vacuolar acidification prevents the escape of bacteria from DVs and subsequent infection of the host macronucleus [37]. Infectious forms of *H. obtusa* escape from early DVs (DV-II) and subsequently infect the host macronucleus, whereas escape does not occur from last-stage DVs (DV-IV). Fok et al. (1982) [38] demonstrated that the pH of DV decreases during vacuole maturation and reaches approximately pH 3 in DV-II, the most acidic stage of this process. Together, these observations are consistent with the possibility that a decrease in the pH of the DV from near-neutral conditions to moderately acidic conditions may trigger the escape of infectious forms of *H. obtusa*, whereas stronger acidification (approximately pH 3) may inhibit escape. Similarly, in bacterial systems, such as *Burkholderia*, acidic environments are required for the expression of virulence factors, and phagosome alkalinization suppresses the infection processes [39,40]. Taken together, these findings suggest that vacuolar acidification functions not only in digestion but also as a conserved regulatory checkpoint governing the intracellular fate of diverse microorganisms. In *P. tritobursaria*, the delayed escape of *Chlorella* sp. from DVs observed under NH_4_Cl treatment is consistent with the possibility that DV acidification may be associated with a regulatory cue controlling escape from DVs. Whether a certain degree of acidification is required to trigger the escape of *Chlorella* sp. or whether more neutral conditions favor escape remains unclear. Nevertheless, the present results indicate that alteration of DV acidification significantly affects the timing of algal escape and, consequently, the transition from a prey state to a symbiotic state. However, because NH_4_Cl affects multiple cellular processes, the present results do not demonstrate a direct causal relationship but rather indicate that acidification is closely associated with this process. Notably, the digestion of *Chlorella* sp. was not completely suppressed in the NH_4_Cl-treated cells, as evidenced by the appearance of brown, degraded algae in both the control and treated groups (Figure 3). This suggests that the experimental conditions used here (20 mM NH_4_Cl for 40 min) do not fully block digestive processes.

### 4.3. Mechanisms Underlying Increased Symbiosis Establishment Rates Following NH_4_Cl Treatment

Despite only partial inhibition of digestion, NH_4_Cl treatment significantly increased the symbiosis establishment rate at 24 h (Figure 4). Importantly, this increase cannot be solely explained by the avoidance of digestion, suggesting a more complex mechanism underlying the establishment of symbiosis. In other systems, the effects of phagosomal pH modulation vary depending on the physiological properties of microorganisms. For example, alkalinization enhances the intracellular survival of *Bordetella pertussis* but reduces the survival of the more acid-tolerant *B. bronchiseptica* [41]. Similarly, alkalinization of lysosomal compartments affects the intracellular growth dynamics of *Cryptococcus neoformans* [42]. These findings indicate that pH changes influence microbial fate in a context-dependent manner. Previous studies on the *P. tritobursaria*–*Chlorella* sp. symbiosis have proposed that multiple sequential events, including ingestion, DV acidification, lysosomal fusion, escape from the DV, and differentiation into the PV membrane, act as checkpoints that determine the establishment of symbiosis [23,24]. Therefore, alterations in the timing or efficiency of these processes are expected to influence the overall success of symbiosis. We propose several possible explanations for the increased symbiosis establishment rates observed under NH_4_Cl treatment. First, suppression of DV acidification may prolong the residence time of *Chlorella* sp. within DVs, thereby increasing the probability of survival. Second, an extended residence time may provide time for the physiological adaptation of algae. Such adaptations may involve changes in the surface properties of algae. Ultrastructural studies have suggested that the cell wall properties of *Chlorella* sp. contribute to symbiosis competence [43], raising the possibility that time-dependent modifications of the algal surface occur during vacuolar residence. Third, we speculate that host *P. tritobursaria* cells may utilize this extended period to selectively retain suitable symbionts. Taken together, these results suggest that transient suppression of DV maturation delays the progression of digestive processes, thereby shifting the balance from digestion to symbiosis and increasing the likelihood of successful establishment of symbiosis.

Interestingly, informal preliminary observations suggest that NH_4_Cl treatment may have little effect on already established symbiosis. These observations should be interpreted cautiously because they were not obtained through a controlled experimental design. Specifically, when algae-bearing *P. tritobursaria* cells from a long-term laboratory culture that had maintained stable symbiosis for more than 14 years were treated with 30 mM NH_4_Cl for up to 3 d, no apparent detachment or enhanced digestion of the symbiotic algae was observed. The remarkable stability of this long-term symbiotic association contrasts with the marked effects of NH_4_Cl during the early stages of symbiosis establishment, where intracellular algal dynamics are altered and symbiosis establishment rates increase. These observations suggest that DV acidification primarily functions during the initial stages of symbiosis establishment, influencing whether ingested algae are digested or retained. Once algal cells become enclosed within the PV and stably localized beneath the host cortex, the maintenance of symbiosis appears to be largely independent of DV-mediated processes and is less sensitive to changes in vacuolar pH.

**Figure 5:**
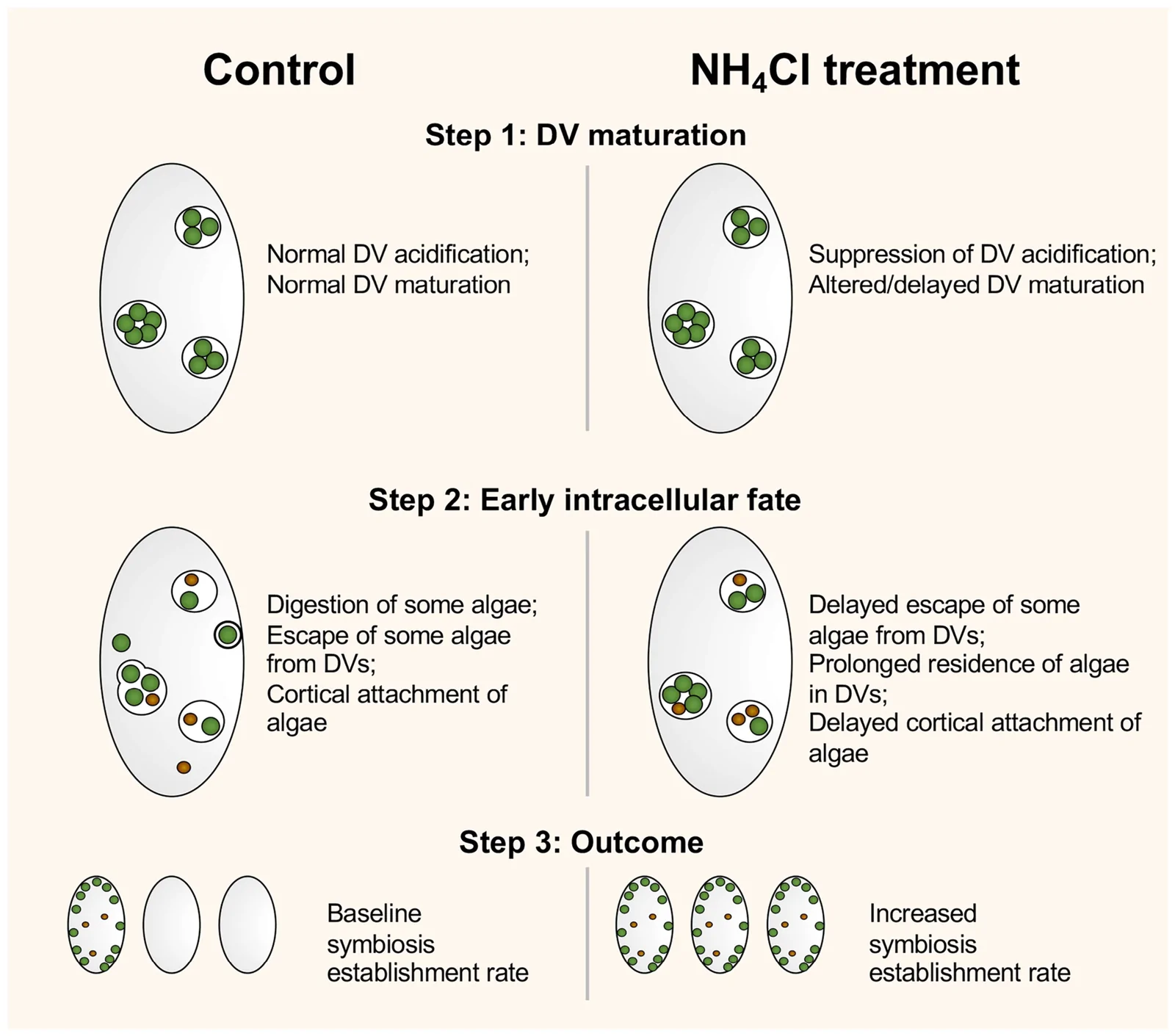
Proposed model for the effects of NH_4_Cl treatment on DV maturation and symbiosis establishment in *P. tritobursaria*. NH_4_Cl treatment suppresses DV acidification and may prolong the residence time of some algal cells within DVs, thereby increasing the probability of symbiosis establishment. Green and brown circles represent undigested and digested *Chlorella* sp. cells, respectively.

Based on the present results, we propose a model for the effects of NH_4_Cl treatment on the establishment of symbiosis (Figure 5). In control cells, ingested algal cells undergo normal DV acidification and maturation, resulting in the digestion of some algal cells, whereas others escape from DVs, attach beneath the host cortex, and establish symbiosis. In contrast, NH_4_Cl treatment suppresses DV acidification and alters or delays DV maturation, leading to prolonged residence of algae within DVs and a delayed escape into the host cytoplasm. Consequently, the progression of algae to the cortical attachment stage was also delayed. Although digestion is not completely inhibited, these changes may increase the probability of successful symbiosis establishment, resulting in a higher symbiosis establishment rate. These findings suggest that the efficiency of symbiosis establishment is determined not only by whether digestion occurs but also by the timing and progression of DV maturation and subsequent intracellular events. Future studies should quantitatively evaluate DV residence time, escape frequency, and cortical localization of algae to further clarify the relationship between DV maturation dynamics and symbiosis establishment.

### 4.4. Methodological Implications and Future Perspectives

Ammonium chloride (NH_4_Cl) and ammonium ions (NH_4_^+^) are known to inhibit lysosomal function by elevating the intralysosomal pH in a wide range of organisms [44,45]. In *Paramecium*, NH_4_Cl is also likely to act on acidosomes and/or DV membranes, thereby reducing the acidification rate. However, it remains unclear whether NH_4_Cl directly promotes the establishment of symbiosis or whether the observed increase in the symbiosis establishment rate is an indirect consequence of impaired acidification. This question cannot be conclusively resolved based on the results of the present study. This study provides a useful methodological framework for manipulating DV acidification in *P. tritobursaria.* In particular, the use of Congo red-stained yeast cells enabled reliable visualization of DV acidification in *P. tritobursaria*. Other tested indicators, such as bromophenol blue and bromocresol green, failed to stain yeast cells effectively, highlighting the difficulty of selecting appropriate probes for this system. Therefore, the successful application of Congo red was important for establishing an experimental system to monitor DV acidification in *P. tritobursaria*. Notably, the pharmacological inhibition of DV acidification has not been systematically studied in *P. tritobursaria*. The ability to reversibly control vacuolar acidification provides a powerful tool for time-resolved analysis of vacuole maturation and the establishment of symbiosis. However, the use of NH_4_Cl alone limits mechanistic interpretation. Future studies should incorporate additional pharmacological inhibitors used by Fok et al. (1987) [28], such as protonophores (e.g., FCCP), ionophores (e.g., monensin), and lysosomotropic agents (e.g., chloroquine), along with quantitative pH measurements using fluorescent probes or ratiometric imaging techniques.

The concentration of NH_4_Cl used in this study was selected on the basis of preliminary observations evaluating the effects of NH_4_Cl on *P. tritobursaria* cell viability. Although a systematic dose–response analysis was beyond the scope of the present study, higher NH_4_Cl concentrations were associated with increased cellular toxicity during prolonged exposure. Therefore, 20 mM NH_4_Cl was adopted as a practical concentration for the transient inhibition of DV acidification. Our preliminary observations further suggested that the duration of NH_4_Cl treatment influenced the time required for recovery of DV acidification after washing. This implies that manipulation of treatment duration may allow a fine-scale dissection of the temporal sequence linking DV acidification, digestion, and symbiosis establishment. Because transient NH_4_Cl treatment significantly increased the symbiosis establishment rate in the present study, modulation of DV acidification may provide a useful strategy for improving the establishment efficiency of artificial host–alga associations. Although further studies are required to determine whether this effect is broadly applicable to other algal strains, experimental manipulation of DV maturation may represent a practical strategy for enhancing the establishment of artificial endosymbiosis in future studies. This approach could facilitate the introduction of beneficial algal partners into aposymbiotic hosts and may contribute to the development of engineered symbiotic systems with improved biological functions. Regardless of the precise molecular mechanism, our results clearly demonstrate that transient inhibition of DV acidification alters the balance between digestion and symbiosis, highlighting the importance of DV maturation dynamics in the establishment of endosymbiosis. The increased symbiosis establishment rate may reflect the prolonged residence of ingested algae within DVs, thereby increasing their probability of survival and providing additional time for physiological adaptation before stable symbiosis is established. Nevertheless, the precise mechanism underlying the effects of NH_4_Cl remains unresolved. These findings suggest that host digestive processes may act as an important checkpoint in determining whether ingested microorganisms are digested as prey or retained as symbionts. Such mechanisms may have contributed to the evolutionary transition from prey capture to stable intracellular symbiosis. Future studies should determine the relative contributions of pH neutralization, membrane trafficking, and vacuole maturation processes to the enhancement of symbiosis establishment observed in this study. In addition, molecular analyses of host signaling pathways and vacuolar maturation markers will be important for elucidating the cellular mechanisms underlying NH_4_Cl-mediated enhancement of symbiosis establishment.

## Data Availability

The original contributions presented in this study are included in the article/supplementary material. Further inquiries can be directed to the corresponding author.

## Supplementary Materials

The following supporting information can be downloaded at https://www.mdpi.com/article/10.3390/microorganisms14081742/s1, Figure S1: Comparison of pH indicator staining in yeast cells and their uptake by alga-free *P. tritobursaria*. Yeast cells stained with bromophenol blue (A,B), bromocresol green (C,D), and Congo red (E,F). Panels (A,C,E) show stained yeast cells before uptake, whereas panels (B,D,F) show yeast cells after uptake by the alga-free *P. tritobursaria*. Scale bars: 10 μm (A,C,E); 20 μm (B,D,F); Figure S2: Experimental design for the inhibition of DV acidification by NH_4_Cl treatment; Figure S3: Experimental design for testing the effect of NH_4_Cl on the establishment of symbiosis; Table S1: The raw data supporting Figure 2 and Figure 4.

## Abbreviations

The following abbreviations are used in this manuscript:

DIC	Differential interference contrast
DV	Digestive vacuole
MDS	Modified Dryl’s solution
NH_4_Cl	ammonium chloride
PV	Perialgal vacuole
